# Favourable effects of consuming a Palaeolithic-type diet on characteristics of the metabolic syndrome: a randomized controlled pilot-study

**DOI:** 10.1186/1476-511X-13-160

**Published:** 2014-10-11

**Authors:** Inge Boers, Frits AJ Muskiet, Evert Berkelaar, Erik Schut, Ria Penders, Karine Hoenderdos, Harry J Wichers, Miek C Jong

**Affiliations:** Department Nutrition and Health, Louis Bolk Institute, Hoofdstraat 24, Driebergen, LA 3972 the Netherlands; Department of Clinical Psycho-Neuro-Immunology, University of Gerona, Gerona, Spain; Department of Laboratory Medicine, University Medical Centre Groningen, Groningen, the Netherlands; Scriptum Communication about Nutrition, IJsselstein, the Netherlands; Food and Biobased Research, Wageningen University & Research centre, Wageningen, the Netherlands; Department of Health Sciences, Mid Sweden University, Sundsvall, Sweden; National Information and Knowledge Centre on Integrative Medicine (NIKIM), Amsterdam, the Netherlands

**Keywords:** Palaeolithic diet, Prevention, Metabolic syndrome, Weight loss correction

## Abstract

**Background:**

The main goal of this randomized controlled single-blinded pilot study was to study whether, independent of weight loss, a Palaeolithic-type diet alters characteristics of the metabolic syndrome. Next we searched for outcome variables that might become favourably influenced by a Paleolithic-type diet and may provide new insights in the pathophysiological mechanisms underlying the metabolic syndrome. In addition, more information on feasibility and designing an innovative dietary research program on the basis of a Palaeolithic-type diet was obtained.

**Methods:**

Thirty-four subjects, with at least two characteristics of the metabolic syndrome, were randomized to a two weeks Palaeolithic-type diet (*n =* 18) or an isoenergetic healthy reference diet, based on the guidelines of the Dutch Health Council (*n =* 14). Thirty-two subjects completed the study. Measures were taken to keep bodyweight stable. As primary outcomes oral glucose tolerance and characteristics of the metabolic syndrome (abdominal circumference, blood pressure, glucose, lipids) were measured. Secondary outcomes were intestinal permeability, inflammation and salivary cortisol. Data were collected at baseline and after the intervention.

**Results:**

Subjects were 53.5 (SD9.7) year old men (*n =* 9) and women (*n =* 25) with mean BMI of 31.8 (SD5.7) kg/m^2^. The Palaeolithic-type diet resulted in lower systolic blood pressure (−9.1 mmHg; *P* = 0.015), diastolic blood pressure (−5.2 mmHg; *P* = 0.038), total cholesterol (−0.52 mmol/l; *P* = 0.037), triglycerides (−0.89 mmol/l; *P* = 0.001) and higher HDL-cholesterol (+0.15 mmol/l; *P* = 0.013), compared to reference. The number of characteristics of the metabolic syndrome decreased with 1.07 (*P* = 0.010) upon the Palaeolithic-type diet, compared to reference. Despite efforts to keep bodyweight stable, it decreased in the Palaeolithic group compared to reference (−1.32 kg; *P* = 0.012). However, favourable effects remained after post-hoc adjustments for this unintended weight loss. No changes were observed for intestinal permeability, inflammation and salivary cortisol.

**Conclusions:**

We conclude that consuming a Palaeolithic-type diet for two weeks improved several cardiovascular risk factors compared to a healthy reference diet in subjects with the metabolic syndrome.

**Trial registration:**

Nederlands Trial Register NTR3002

## Background

The metabolic syndrome (MetS) is a clustering of symptoms that usually derive from a combination of over nutrition and a sedentary lifestyle [1]. Because of the worldwide rapidly increasing prevalence of the MetS and its high risk of progression to type 2 diabetes mellitus (DM2) [2, 3] and cardiovascular disease (CVD) [4], preventive measures are urgently needed. Lifestyle-induced insulin resistance and chronic systemic low grade inflammation may be central in the pathophysiological cascade towards the MetS [5]. Although dietary management and lifestyle modifications are the cornerstones in the treatment and prevention of metabolic disorders, specific guidelines for the MetS have not yet been established. There is considerable evidence that the dietary composition may favourably affect the components of the MetS. Various authors have pointed at the discordance between our contemporary diet and that of our Palaeolithic ancestors, which has shaped our core metabolism and physiology during the past 2.5 million years. Evolution predicts that such diets may also be optimal for prevention and treatment of metabolic disorders associated with obesity, DM2, CVD and insulin resistance [6–13]. Previously reported studies with Palaeolithic-type diets were conducted with healthy subjects [14, 15] or patients with CVD [16], DM2 [17] or obesity [18, 19]. There are no studies on the effects of a Palaeolithic-type diet targeting subjects with the MetS. The conducted trials showed that Palaeolithic-type diets may effectively lower bodyweight, waist circumference and BP [14], lower serum lipids [18] and improve insulin response in healthy volunteers within less than three weeks [15]. A major limitation of these three studies was the lack of a reference group. Controlled studies in patients with ischaemic heart disease [16], DM2 [17] or obese postmenopausal women [19], without correction for possible weight loss, showed a larger improvement of various CVD risk factors after consuming a Palaeolithic-type diet. Consequently, it is uncertain whether any of the positive health effects in these studies could also be on account of the accompanying weight loss as opposed to the composition of the Palaeolithic-type diet *per se*.

The main goal of the current pilot study was to compare metabolic effects of a Palaeolithic-type diet with those of a healthy reference diet, independent of weight loss, in subjects with the MetS. Next we studied outcome variables that might become favourably influenced by a Palaeolithic-type diet, and thereby provide new insights into the pathophysiological mechanisms underlying the MetS and CVD. The third goal was to obtain more information on feasibility and how to design and develop an innovative dietary research program on the basis of a Palaeolithic-type diet.

## Methods

### Study design

This was a stratified (men/women), randomized controlled single-blinded pilot study conducted in the Netherlands. Subjects with characteristics of the MetS were randomized to a two weeks dietary intervention with either a Palaeolithic-type diet or a healthy reference diet. Emphasis was put on the prevention of weight loss during the intervention. The study took place at the Louis Bolk Institute in Driebergen, the Netherlands, in October-December 2011. Laboratory measurements were performed in the Diakonessenhuis, Zeist, the Netherlands.

### Subjects

Eligible subjects were 18–70 years old adults who gave written consent and had at least two of the following characteristics of the MetS [20]: 1. Central obesity (waist circumference ≥102 cm for men and ≥88 cm for women) 2. Elevated triglycerides ≥1.7 mmol/l 3. Reduced HDL-cholesterol <1.0 mmol/l for men and <1.3 mmol/l for women) 4. Raised BP ≥130/85 mmHg or BP medication and 5. Elevated fasting plasma glucose ≥5.6 mmol/l. Exclusion criteria were DM2, CVD, smoking, systolic BP > 180 mmHg, hypoglycaemic medication, pregnancy and severe internal or psychiatric disease. The study was approved by the Medical Ethics Committee Wageningen University and conducted according to the principles expressed in the Declaration of Helsinki.

### Study settings

Subjects were recruited in September-October 2011 through advertisements in local newspapers and posters in primary health care units. After telephone contact a first selection was made and subjects were enrolled during a screening visit by the research physician. It was not known to the subjects whether any of the diets would be superior to the other (single-blinded). After randomization, subjects of the different diet groups could not communicate with each other. Before and directly after the intervention period subjects visited the Diakonessenhuis in Zeist for physical measurements, OGTT and blood sampling. All meals were delivered at their homes free of charge by a catering service.

### Dietary interventions

The Palaeolithic-type diet intervention was based on anthropological Palaeolithic research [21, 22] with a concern for feasibility in modern times. It was based on lean meat, fish, fruit, leafy and cruciferous vegetables, root vegetables, eggs and nuts. Dairy products, cereal grains, legumes, refined fats, extra salt and sugar were not part of it. The reference diet was based on the guidelines for a healthy diet of the Dutch Health Council [23–31]. Both diets were designed as seven consecutive daily menus (breakfast, lunch, dinner and snacks) and provided on the basis of an isoenergetic intake of 8 700 kJ. Although coffee and tea were not part of a Palaeolithic-type diet, subjects were allowed to drink, in view of possible withdrawal symptoms, up to two cups of coffee or black tea per day. Any medication was continued at the same dosages. Additional detailed information on the two diets is summarized in Table 1.

**Table 1:** **Nutrient and caloric composition of the dietary intervention programs**

Nutrient	Palaeolithic^a^		Reference^a^		RDA^b^	
	En%		En%		En%	
Energy (kJ)	8 703		8 690			8 374
Protein (en%),(g)	24	123	17	91	10-25	
vegetable protein (g)	22		39		none	
animal protein (g)	101		52		none	
Carbohydrate (en%),(g)	32	164	50	261	40-70	
mono/disaccharides (g)	132		109		none	
Fat (en%),(g)	41	94	29	68	20-40	
saturated (en%),(g)	10	24	9	21	<10	
monounsaturated (g)	44		26.5		none	
polyunsaturated (g)	19		14.6		none	
linoleic acid (g)	14		11		4-6	
EPA (mg)	640		210		EPA + DHA: 450	
DHA (mg)	950		360		EPA + DHA: 450	
Fibre (en%),(g)	3	34	2.7	28	30-40	
Sodium (mg)	2 194		2 121		<2 400	
Potassium (mg)	5 859		3 932		4 700	
Calcium (mg)	575		1 181		1 000	
Magnesium (mg)	494		415		250-300	
Iron (mg)	16.4		12.6		8-15	
Selenium (mg)	110		50		50-150	
Zinc (mg)	13.6		12.0		9-10	
Phosphorus (mg)	1 661		1 729		700-1 400	
Copper (mg)	2.0		1.4		1.5-3.5	
Iodine (mcg)	138		219		150	
Vitamin A (mcg)	1 317		705		700-900	
Vitamin B1 (mg)	1.5		1.0		1.1	
Vitamin B2 (mg)	1.4		1.7		1.1-1.5	
Vitamin B3 (mg)	35		19		13-17	
Vitamin B6 (mg)	2.8		1.7		1.5	
Vitamin B12 (mcg)	11.5		4.2		2.8	
Vitamin C (mg)	264		142		70	
Vitamin D (mcg)	4.3		3.7		3.3	
Folate (mcg)	398		331		300	

*Abbreviation: En%*, energy percentage.

^a^Calculations were based on the Dutch NeVo table [30].

^b^Recommended Dietary Allowances were based on recommendation of the Dutch Health Council [22–29].

### Prevention of weight loss

Before the intervention all subjects recorded their usual diet using a food record diary. This diary was used by the dietician to adjust diets to individual energy demand by providing (additional) program-related snacks to prevent weight loss within the intervention period. Bodyweight was measured every second day. A daily bodyweight fluctuation of 2 kg was considered acceptable. Next to keeping bodyweight stable, importance was emphasized of keeping their usual exercise level during the intervention.

### Compliance

To ensure compliance to the intervention, all subjects were requested to keep records of the food consumed. In addition, each subject was encouraged every other day by telephonic contact with their personal coach to complete all meals and to discuss their progress, bodyweight fluctuation, possible physical and psychological discomforts or adverse events. A total of six personal coaches (each coaching 5–6 subjects) were involved during the study, who could be reached by the subjects for any questions concerning catering, daily recording or measurements.

### Outcomes

Primary endpoints were glucose tolerance measured by the oral glucose tolerance test (OGTT) [32] and the characteristics of the MetS (waist circumference, systolic/diastolic BP, lipids, fasting glucose). Additional analyses comprised intestinal permeability, inflammation parameters (hsCRP, TNFα) and a stress parameter (salivary cortisol). Tolerability was measured by analysing the adverse events reported during the study and by performing blood tests for haematological indices and liver and kidney functions.

### Measurements

Systolic and diastolic BP, from which the mean values were calculated, were measured twice after at least 10 min of sitting using an automated BP measuring device (OMRON M6 Comfort, OMRON Healthcare Co. Ltd.). Bodyweight was measured using a digital Seca 803 scale. For the OGTT subjects ingested a solution containing a 75 g glucose load. Plasma glucose and insulin concentrations were measured at 0 (baseline), 30, 60 and 120 min after ingestion. AUCs for glucose and insulin were calculated using the trapezoidal method. In addition HOMA_IR_ and the ratio between triglycerides and HDL-cholesterol were determined as measures of insulin sensitivity. Urine was collected for assessment of intestinal permeability, which was measured using the differential sugar absorption test (DSAT). After an overnight fast, the subjects ingested a test solution containing 5 g lactulose, 2 g mannitol and 5 g D-xylose in 100 ml water. All urine portions passed in the subsequent five hours were collected in a plastic container containing 0.5 ml 20% chlorhexidine as a preservative. Results were expressed as the urinary lactulose:mannitol ratio. Saliva was collected by Salivette® devices for assessment of the diurnal cortisol slope. Subjects had to chew on the cotton swab for 30–60 seconds. Sampling was performed at wake time, 30–40 minutes after waking (peak time), 17.00 h and bedtime [33]. A flattening slope of the diurnal cortisol curve is related to low-grade inflammation, insulin resistance and the MetS [34–36]. At the end of the intervention, an evaluation form on feasibility and motivational aspects was filled out by all subjects.

### Statistical methods

For this pilot design a sample size of 15 subjects per group was estimated to be sufficient, because in previous studies with Palaeolithic-type diets [15, 21] the same number of subjects showed positive changes in BP, glucose tolerance, lipids and inflammation parameters within two weeks. To allow for an estimated 20% drop out, the total number of subjects was set at 36. Subjects were stratified into two groups according to gender and subsequently randomized using separate randomization lists as generated by the Random Allocation Software Program employing a random block size of 6 to guarantee balanced allocation. The study monitor generated the random allocation sequence and assigned subjects to interventions. Descriptive statistics were calculated with regard to demographical and categorical data and mean scores and SDs of the numerical variables using SPSS (version 17.0). Evaluation occurred according to intention-to-treat. Mixed models were used to analyse between groups. This model included intervention, gender, intervention by gender, baseline BMI and the baseline value of the specific outcome variable as fixed effects, the intercept and subjects identification number as random effects. Pair wise comparisons between the two diets were made using the adjusted data. For these differences 95% confidence intervals were calculated. Two-way paired t-tests were used to analyse within-subject changes per group according to per-protocol.

## Results

**Figure 1:**
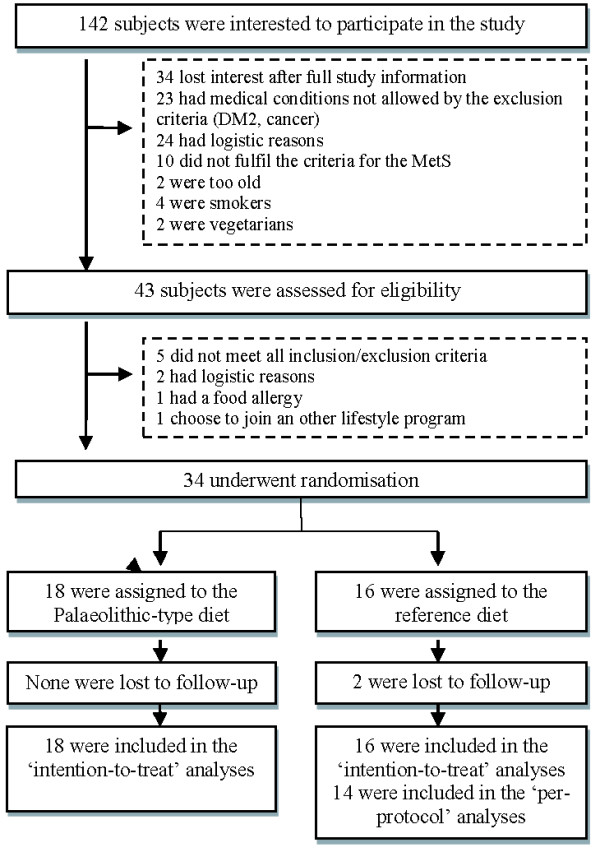
**Flow diagram of the progress through enrolment, intervention allocation, follow-up, and data analysis.**

The courses of inclusion and exclusion are shown in Figure 1. A total of 34 subjects were randomized: 18 were allocated to the Palaeolithic group and 16 to the reference group. Two subjects (2/16; 12.5%) dropped out from the reference group for personal reasons. There were only a few at random missing cortisol data, attributable to some subjects who forgot to take a saliva sample.

### Baseline data

Tables 2, 3 and 4 show the baseline demographic and clinical characteristics of all subjects. Baseline data differed between both groups for BMI and number of characteristics of the MetS. No differences were found for coffee, tea and alcohol consumption, or the amount of exercise and stress factors between the intervention groups. All outcome variables exhibited normal distributions. Exception was the diurnal slope of cortisol showing a skewed distribution.

**Table 2:** **Baseline demographic and clinical characteristics**

Variable	Palaeolithic (***n***18)		Reference (***n***16)		P*
	Mean or %	SD	Mean or %	SD	
**Demographic**					
Gender: women (%)	72.2		75.0		0.86
Age (year)	52.0	10.2	55.4	9.0	0.32
Race					0.13
Caucasian (%)	100		87.5		
Asian (%)	0		12.5		
**Characteristics of the MetS** (*n*), range 0-5	3.7	1.1	2.7	1.3	0.02
(1) Abdominal circumference ♂ ≥102 cm, ♀ ≥88 cm (%)	94.4		100.0		0.35
(2) Triglycerides ≥1.7 mmol/l (%)	38.9		18.8		0.21
(3) HDL-cholesterol ♂ <1.0 mmol/l , ♀ <1.3 mmol/l (%)	66.7		12.5		0.00
(4) BP ≥130/85 mmHg or BP medication (%)	83.3		75.0		0.56
(5) Glucose *fasting* ≥5.6 mmol/l (%)	77.8		43.8		0.04

*P values between the two intervention groups at baseline.

**Table 3:** **Summary results for each intervention group**

Variable		Palaeolithic (***n***18)		Reference (***n***16)		***P*** ^a^	Difference^b^	***P*** ^c^
		Mean	SD	Mean	SD			
**Anthropometric**								
Bodyweight (kg)							−1.3 (−2.3; −0.3)	0.01
	*Baseline*	98.0	18.2	86.0	14.2	0.04		
	*2 weeks*	95.3	17.5	84.3	12.5			
BMI (kg/m^2^)							−0.5 (−0.9; −0.1)	0.01
	*Baseline*	33.7	5.9	29.8	4.9	0.05		
	*2 weeks*	32.8	5.8	29.7	5.2			
Abdominal circumference (cm)							−0.4 (−2.4; 1.6)	0.69
	*Baseline*	114.7	11.5	107.7	9.4	0.06		
	*2 weeks*	111.6	12.3	104.7	8.7			
Systolic BP (mmHg)							−9.1 (−16.2; −1.9)	0.02
	*Baseline*	131	15	134	15	0.48		
	*2 weeks*	122	10	129	14	·		
Diastolic BP (mmHg)							−5.2 (−10.0; −0.3)	0.04
	*Baseline*	87	9	86	13	0.75		
	*2 weeks*	79	6	83	9	·		
Characteristics of the MetS (*n*),							−1.1 (−1.9; −0.3)	0.01
range 0-5	*Baseline*	3.7	1.1	2.7	1.3	0.02		
	*2 weeks*	2.7	1.0	2.9	1.2			
**Glucose tolerance and insulin sensitivity**								
Glucose *fasting* (mmol/l)							−0.1 (−0.5; 0.3)	0.68
	*Baseline*	6.1	0.8	5.8	0.7	0.34		
	*2 weeks*	5.7	0.8	5.5	0.8			
Insulin *fasting* (mU/l)							−2 · 0 (−5.0; 1.1)	0.20
	*Baseline*	11.9	5.5	10.2	6.5	0.40		
	*2 weeks*	9.2	4.9	9.5	5.3			
HOMA_IR_^d^							−0.5 (−1.4; 0.4)	0.28
	*Baseline*	3.3	1.7	2.7	1.8	0.32		
	*2 weeks*	2.4	1.6	2.4	1.3			
AUC *glucose* (mmol/l x min)							−27.7 (−156.6; 101.1)	0.66
	*Baseline*	263	208	249	162	0.83		
	*2 weeks*	245	199	262	216			
AUC *insulin* (mU/l x min)							−1 342 · (−415; 3 099)	0.13
	*Baseline*	8 791	6 200	6 270	3 619	0.16		
	*2 weeks*	6 873	2 692	6 955	3 365			
TG:HDL-C (mol/mol)							−0.9 (−1.3; −0.5)	0.00
	*Baseline*	1.7	1.6	0.9	0.6	0.07		
	*2 weeks*	0.9	0.7	1.1	0.7			
**Lipids**								
TC (mmol/l)							−0.5 (−1.0; −0.0)	0.04
	*Baseline*	5.7	1.0	6.1	1.4	0.33		
	*2 weeks*	5.0	0.9	6.0	1.2			
HDL-C (mmol/l)							0.2 (0.0; 0.3)	0.01
	*Baseline*	1.3	0.4	1.6	0.4	0.06		
	*2 weeks*	1.3	0.4	1.4	0.4			
LDL-C (mmol/l)							−0.1 (−0.5; 0.3)	0.56
	*Baseline*	3.5	0.7	3.9	1.4	0.27		
	*2 weeks*	3.2	0.8	3.9	1.1			
TG (mmol/l)							−0.9 (−1.3; −0.5)	0.00
	*Baseline*	1.9	1.4	1.3	0.6	0.10		
	*2 weeks*	1.0	0.6	1.4	0.6			
TC:HDL-C (mol/mol)							−1.2 (−1.9; −0.4)	0.00
	*Baseline*	4.6	1.5	3.5	2.1	0.11		
	*2 weeks*	4.0	1.3	4.5	1.5			
**Inflammation**								
hsCRP (mg/l)							0.1 (−2.2; 2.3)	0.96
	*Baseline*	4.4	3.4	2.3	3.1	0.07		
	*2 weeks*	4.6	3.1	3.0	4.5			
TNFα (pg/ml)							−0.6 (−1.7; 0.6)	0.32
	*Baseline*	3.9	1.8	4.3	3.0	0.65		
	*2 weeks*	4.0	1.6	4.3	3.1			
**Intestinal permeability**								
Lactulose:mannitol (mol/mol), *in 5 h urine portion*							−0.007 (−0.023; 0.008)	0.35
	*Baseline*	0.029	0.017	0.024	0.021	0.66		
	*2 weeks*	0.024	0.013	0.033	0.025			
**Stress parameter**								
Diurnal cortisol slope, *wakeup-bedtime*							−0.08 (−0.36; 0.20)	0.54
	*Baseline*	−0.41	−0.30;-0.72^e^	−0.38	−0.30;-0.70^e^	0.43		
	*2 weeks*	−0.40	−0.22;-0.54^e^	−0.57	−0.28;-0.62^e^			

*Abbreviations*: *TG* triglycerides, *HDL-C* HDL-cholesterol, *TC* total cholesterol, *LDL-C* LDL-cholesterol.

Mean values and standard deviations, intention-to-treat.

^a^ *P* values between the two intervention groups at baseline.

^b^Difference = Palaeolithic-type diet – reference diet. Values are 95% CI.

^c^ *P* values between the two intervention groups after the intervention.

^d^For HOMA_IR_ the following equation was used: HOMA_IR_ = (fasting insulin mmol/l x fasting glucose mU/l)/22.4.

^e^Data are median (25st;75st percentile).

**Table 4:** **Summary results for each intervention group – tolerability**

Variable		Palaeolithic (***n***18)		Reference (***n***16)		***P*** ^a^	Difference^b^	***P*** ^c^
		Mean	SD	Mean	SD			
**Haematology**								
Hb (g/l)							−1.6 (−6.4;3.2)	0.59
	*Baseline*	145.0	11.3	141.8	8.1	0.44		
	*2 weeks*	145.0	11.3	143.4	9.7			
Ht (l/l)							0.00 (−0.01;0.01)	0.93
	*Baseline*	0.43	0.03	0.42	0.03	0.40		
	*2 weeks*	0.44	0.03	0.42	0.03			
RBC (x10E^12^/l)							−0.0 (−0.2;0.1)	0.58
	*Baseline*	4.8	0.3	4.8	0.3	0.28		
	*2 weeks*	4.8	0.3	4.7	0.4			
WBC (x10E^9^/l)							−1.0 (−1.7;-0.2)	0.02
	*Baseline*	7.2	1.9	7.1	1.7	0.88		
	*2 weeks*	6.3	1.2	7.0	1.8			
Platelets (x10E^9^/l)							−15.5 (−35.7;4.7)	0.13
	*Baseline*	253.6	44.4	255.1	70.5	094		
	*2 weeks*	240.2	51.5	262.5	72.8			
**Liver and kidney function**								
ASAT (U/l)							0.9 (−3.1;4.9)	0.63
	*Baseline*	27.6	7.0	26.3	6.8	0.57		
	*2 weeks*	30.6	6.8	25.3	6.6			
ALAT (U/l)							1.9 (−5.5;9.2)	0.61
	*Baseline*	35.1	16.8	25.9	11.9	0.08		
	*2 weeks*	38.2	17.7	25.1	10.2			
GGT (U/l)							−9.5 (−17.7;-1.3)	0.03
	*Baseline*	27.3	11.1	38.9	39.3	0.24		
	*2 weeks*	21.9	8.1	37.6	40.1			
AP (U/l)							−4.0 (−9.4;1.5)	0.15
	*Baseline*	79.3	22.1	70.0	13.3	0.15		
	*2 weeks*	74.1	21.3	70.1	11.5			
Creatinine (μmol/l)							−3.1 (−9.8;3.6)	0.36
	*Baseline*	71.2	11.4	76.1	13.0	0.25		
	*2 weeks*	75.6	13.8	79.0	14.6			
Urea (mmol/l)							−0.0 (−0.8;0.8)	0.92
	*Baseline*	4.9	1.3	5.1	1.2	0.58		
	*2 weeks*	5.7	1.6	6.0	1.3			
**Other laboratory parameters**								
*Urine*								
Sodium (mmol/h)							−0.5 (−1.7;0.8)	0.46
	*Baseline*	6.3	0.2	6.7	2.9			
	*2 weeks*	3.8	2.7	3.4	1.0			
Potassium (mmol/h)							0.1 (−1.1;1.2)	0.91
	*Baseline*	3.5	1.3	4.0	1.6			
	*2 weeks*	3.8	1.5	3.4	1.3			
Magnesium (mmol/h)							−0.04 (−0.07;-0.01)	0.03
	*Baseline*	0.14	0.06	0.14	0.06			
	*2 weeks*	0.13	0.08	0.13	0.06			
Calcium (mmol/h)							−0.08 (−0.12;-0.04)	0.00
	*Baseline*	0.13	0.13	0.19	0.20			
	*2 weeks*	0.08	0.05	0.16	0.10			
Sodium:potassium (mmol/mol)							−0.20 (−0.62;0.22)	0.34
	*Baseline*	2.12	1.16	1.78	0.67	0.31		
	*2 weeks*	1.00	0.47	1.15	0.55			
*Serum*								
Homocysteine (μmol/l)							1.05 (−0.44;2.55)	0.16
	*Baseline*	11.7	3.9	12.6	3.0	0.31		
	*2 weeks*	12.9	4.3	12.8	3.1			

*Abbreviations: Ht* Haematocrit, *RBC* Red blood cell count, *WBC* White blood cell count, *ASAT* Aspartate aminotransferase, *ALAT* Alanine amino transferase, *GGT* γ-Glutamyltransferase, *AP* Alkaline phosphatase.

Mean values and standard deviations, intention-to-treat.

^a^ *P* values between the two intervention groups at baseline.

^b^Difference = Palaeolithic-type diet – reference diet (with 95% CI).

^c^ *P* values between the two intervention groups after the intervention.

### Outcomes and estimation

Study subjects were 9 men and 25 women with an average age of 53.5 (SD9.7) years, a mean BMI of 31.8 (SD5.7) and an average number of 3.2 (SD1.3) characteristics of the MetS. Compared to reference, the Palaeolithic group, had lower mean systolic BP (−9.1 mmHg; *P* = 0.015), diastolic BP (−5.2 mmHg; *P* = 0.038), total cholesterol (−0.52 mmol/l; *P* = 0.037) and triglycerides (−0.89 mmol/l; *P* = 0.001) and a higher mean HDL-cholesterol (+0.15 mmol/l; *P* = 0.013). For detailed results see Tables 3 and 4. The number of characteristics of the MetS decreased upon the Palaeolithic-type diet (−1.07 characteristics; *P* = 0.010) compared to reference, resulting in less subjects with the actual MetS according to the definition of the MetS [20] after the intervention. There was a tendency for a larger decrease of AUC insulin in the Palaeolithic group (−1 918 mU/l x min versus +362 mU/l x min in the reference group; *P* = 0.051) using two-tailed unpaired t-tests, however this difference did not remain when adjusted for baseline BMI. In both groups change was observed in waist circumference (Palaeolithic: −3.1 cm; reference: −3.3 cm), fasting glucose (Palaeolithic: −0.9 mmol/l; reference: −0.35 mmol/l) and the ratio between urinary sodium and potassium (Palaeolithic: −1.1; reference: −0.57), however no differences were seen between both groups. Fasting plasma insulin (−2.7 mU/l) and HOMA_IR_ (−0.9) only decreased in the Palaeolithic group (Table 5). The ratio between total cholesterol and HDL-cholesterol (−1.16 mol/mol; *P* = 0.003) and the ratio between triglycerides and HDL-cholesterol (−0.91 mol/mol; *P* = 0.0001) were lowered in the Palaeolithic group compared to reference. Among tolerability outcomes the white blood cell count and γ-glutamyltransferase showed a decline in favour of the Palaeolithic group. Urine analysis showed lower values of magnesium and calcium in the Palaeolithic group compared to reference. Other secondary outcome parameters showed no changes.

**Table 5:** **Differences between baseline and after intervention for both groups**

Variable	Paired Differences								
	All (***n***34)			Palaeolithic (***n***18)			Reference (***n***14)		
	Mean	SD	P*	Mean	SD	P*	Mean	SD	P*
**Anthropometric**									
Abdominal circumference (cm)	−3.2	2.2	0.00	−3.1	2.0	0.00	−3.3	2.4	0.00
Systolic BP (mmHg)	−6.6	9.8	0.00	−8.5	12.0	0.01	−4.2	5.6	0.02
Diastolic BP (mmHg)	−6.0	7.5	0.00	−8.0	8.3	0.0	−3.5	5.6	0.03
**Glucose tolerance and insulin sensitivity**									
Glucose *fasting* (mmol/l)	−0.4	0.5	0.00	−0.4	0.5	0.01	−0.4	0.4	0.00
Insulin *fasting* (mU/l)	−2.1	4.3	0.01	−2.7	5.0	0.03	−1.4	3.2	0.14
HOMA_IR_	−0.7	1.3	0.00	−0.9	1.5	0.03	−0.5	0.9	0.06
AUC *glucose* (mmol/l x min)	−6	141	0.81	−18	170	0.66	9	98	0.73
AUC *insulin* (mU/l x min)	−921	3 565	0.15	−1 918	4 361	0.08	362	1 515	0.39
TG:HDL-C (mol/mol)	−0.4	1.0	0.04	−0.8	1.2	0.01	0.2	0.3	0.08
**Lipids**									
TC (mmol/l)	−0.6	0.7	0.000	−07	0.7	0.00	−0.4	0.5	0.02
HDL-C (mmol/l)	−0.1	0.2	0.001	−0.0	0.1	0.38	−0.2	0.1	0.00
LDL-C (mmol/l)	−0.2	0.5	0.01	−0.3	0.5	0.02	−0.2	0.5	0.18
TG (mmol/l)	−0.5	1.0	0.01	−0.9	1.1	0.00	0.1	0.4	0.63
TC:HDL-C (mol/mol)	0.1	1.2	0.72	−0.5	0.7	0.01	0.9	1.3	0.03
**Inflammation**									
hsCRP (mg/l)	0.3	2.5	0.54	0.2	2.8	0.79	0.4	2.1	0.49
TNFα (pg/ml)	0.2	1.3	0.45	0.1	1.6	0.87	0.3	0.7	0.13
**Intestinal permeability**									
Lactulose:mannitol (mmol/mol)	0.003	0.020	0.3	−0.002	0.020	0.65	0.009	0.012	0.11
**Other laboratory parameters**									
*Urine*									
Sodium:potassium (mmol/mol)	−0.88	0.93	0.00	−1.11	0.97	0.00	−0.57	0.82	0.02
*Serum*									
Homocysteine (μmol/l)	0.8	1.6	0.01	1.2	1.3	0.00	0.3	1.9	0.60

*Abbreviations*: *TG* triglycerides, *HDL-C* HDL-cholesterol, *TC* total cholesterol, *LDL-C* LDL-cholesterol.

Mean values and standard deviations, per-protocol.

*P values for the difference between baseline and after intervention, computed by two-tailed paired t-test, not adjusted.

In line with the protocol, we took efforts to keep bodyweight stable. For nine subjects extra diet-related snacks were necessary due to over 2 kg weight loss, without being hungry (seven in the Palaeolithic group = 38% versus two in the reference group = 14%). Hunger was reported once by three subjects in the Palaeolithic group at the start of the intervention. According to food diaries and coach reports, all subjects were able to complete their dietary programs. At the end of the intervention bodyweight was nevertheless decreased in the Palaeolithic group compared to reference (−1.32 kg; *P* = 0.012). After adding this (unintended) weight loss as a separate fixed effect in a post-hoc analysis we found that favourable effects remained for systolic BP, HDL-cholesterol, triglycerides, the ratio between triglycerides and HDL-cholesterol, the ratio between total cholesterol and HDL-cholesterol and the number of characteristics of the MetS (Table 6).

**Table 6:** **Post-hoc analysis: differences without and with weight loss added to the model**

Variable	Difference^a^	***P*** ^b^	Adjusted difference^c^ ***weight loss added to the model***	***P*** ^b^
**Anthropometric**				
Abdominal circumference (cm)	−0.4 (−2.4;1.6)	0.69	−0.9 (−3.2;1.5)	0.56
Systolic BP (mmHg)	−9.1 (−16.2;-1.9)	0.02	−13.0 (−25.4;-0.6)	0.04
Diastolic BP (mmHg)	−5.2 (−10.0;-0.3)	0.04	−4.9 (−10.7;0.9)	0.09
Characteristics of the MetS (*n*)	−1.1 (−1.9;-0.3)	0.01	−1.0 (−1.8;-0.1)	0.03
**Glucose tolerance and insulin sensitivity**				
Glucose *fasting* (mmol/l)	−0.1 (−0.5;0.3)	0.68	−0.0 (−0.5;0.5)	0.91
Insulin *fasting* (mU/l)	−2.0 (−5.0;1.1)	0.20	−1.4 (−5.1;2.3)	0.44
HOMA_IR_	−0.5 (−1.4;0.4)	0.28	−0.5 (−1.6;0.7)	0.41
AUC *glucose* (mmol/l x min)	−28 (−157;101)	0.66	−107 (−247; 33)	0.13
AUC *insulin* (mU/l x min)	−1 342 (−415; 3 099)	0.13	−484 (−2 503; 1 535)	0.63
TG:HDL-C (mol/mol)	−0.9 (−1.3;-0.5)	0.00	−0.7 (−1.1;-0.3)	0.00
**Lipids**				
TC (mmol/l)	−0.5 (−1.0;-0.0)	0.04	−0.3 (−0.8;0.3)	0.35
HDL-C (mmol/l)	0.2 (0.0;0.3)	0.01	0.15 (0.01;0.29)	0.04
LDL-C (mmol/l)	−0.1 (−0.5;0.3)	0.56	−0.1 (−0.5;0.4)	0.75
TG (mmol/l)	−0.9 (−1.3;-0.5)	0.00	−0.7 (−1.0;-0.3)	0.00
TC:HDL-C (mol/mol)	−1.2 (−1.9;-0.4)	0.00	−1.1 (−2.0;-0.3)	0.01
**Inflammation**				
hsCRP (mg/l)	0.1 (−2.2;2.3)	0.96	0.1 (−2.7;2.8)	0.95
TNFα (pg/ml)	−0.6 (−1.7;0.6)	0.32	−0.7 (−2.0;0.7)	0.31
**Intestinal permeability**				
Lactulose:mannitol (mmol/mol)	−0.07 (−0.023;0.008)	0.35	−0.012 (−0.031;0.008)	0.24
**Stress parameter**				
Diurnal cortisol slope	−0.08 (−0.36;0.20)	0.54	−0.10 (−0.44;0.25)	0.24
*wakeup-bedtime*				

*Abbreviations*: *TG* triglycerides, *HDL-C* HDL-cholesterol, *TC* total cholesterol, *LDL-C* LDL-cholesterol.

Adjusted differences after the intervention between both diets without and with weight loss added to the model.

^a^Difference = Palaeolithic-type diet – reference diet (with 95% CI).

^b^ *P* values between the two intervention groups after the intervention.

^c^Adjusted difference = Palaeolithic-type diet – reference diet (with 95% CI), percentage weight loss added to the model as fixed effect.

### Adverse events

One subject in the reference group reported a moderate adverse event (nausea and diarrhoea) during the intervention which was not likely related to the intervention. Blood analysis concerning haematology and liver and kidney function showed no changes in relation to adverse events (Table 4).

## Discussion

This is the first controlled study on the influence of a Palaeolithic-type diet in subjects with characteristics of the MetS. Over a two week period we found favourable changes in most characteristics of the MetS: a lowering of BP, a change in lipid spectrum and a tendency to higher insulin sensitivity. Despite efforts to keep bodyweight stable, more weight loss was observed in the Palaeolithic group, which is an important outcome of our study. Nevertheless, favourable effects remained after adding weight loss as a separate fixed effect in a post-hoc analysis. No changes were found in specific inflammation parameters, intestinal permeability and the diurnal cortisol slope, possibly explained by the short duration of the dietary intervention or its constant bodyweight design. Indicators for new insights in the pathophysiological mechanisms underlying the MetS and CVD could possibly be found in the lowering effect on white blood cell count and lower levels of urinary calcium and magnesium. The generalizability of this study might be high, because of the broad inclusion criteria, men and women with diverse combinations and characteristics of the MetS.

### Interpretation

Our results are in line with results from earlier research in related target groups. We found a lowering of systolic and diastolic BP, where in uncontrolled studies with healthy volunteers [14, 15] as well as in controlled studies with diabetic patients [17] lowering of BP has also been reported. Contrarily, BP lowering was not observed in controlled studies concerning patients with ischaemic disease [16] and obese postmenopausal women [19]. With respect to lipids, we found positive changes in total cholesterol, triglycerides and HDL-cholesterol in favour of the Palaeolithic group. Previous research also reported positive changes in lipid profile in healthy subjects, diabetic patients or obese women [15, 17–19]. In our study, the ratio between total cholesterol and HDL-cholesterol, which is an important risk factor for CVD employed in clinical practice, was also lowered in the Palaeolithic group compared to reference. In addition, the ratio between triglycerides and HDL-cholesterol, a proxy for insulin resistance, was lowered in the Palaeolithic group compared to reference. Regarding glucose tolerance we did not find significant changes in our study. There was, however, a tendency for a larger decrease of AUC insulin and HOMA_IR_ in the Palaeolithic group. In contrast to our results, glucose tolerance improved within 10–17 days in healthy subjects after a Palaeolithic diet in an uncontrolled study [15], as well as in a controlled study of longer duration (3 months) in patients with ischemic heart disease [16]. In our study we found for both groups a decrease in abdominal circumference of approx. 3 cm, but not as a specific effect of the Palaeolithic-type diet. Earlier findings in 3 months [16, 17] and 6 months [19] controlled studies did report change in abdominal circumference in favour of the Palaeolithic-type diet, in patients with ischemic disease and DM2 [16, 17] or obese postmenopausal women [19]. Also other low-carbohydrate, high-protein or Mediterranean diets have demonstrated to be effective in improving various markers of cardiovascular risk in diabetic patients [37]. Unique for our study was the attempt to find these favourable effects independently from the factor of weight loss. Another notable observation was that characteristics of the MetS can be reduced within a short period in people with cardiovascular risk.

A frequently expressed critique on Palaeolithic-type diets is related to their low calcium content. In our study there was indeed a 50% lower calcium intake compared to the reference diet (Table 1). However, due to higher dietary magnesium intake compared to reference and compensation by lower urinary calcium and magnesium excretion, calcium homeostasis was unlikely to have become compromised.

### Course of study and feasibility

Home catering with frequent coaching was a satisfactory method to monitor the intake and whereabouts of the subjects. Subjects remained motivated and were coached to consume the prescribed food. Problems could be solved adequately resulting in few drop-outs and missing data. Nevertheless, we experienced that delivering fresh foods in time across a wide region of the Netherlands was a logistical challenge. For follow-up studies, attention to catering aspects is of utmost importance. The experienced difficulties in maintaining bodyweight could be part of the positive effects of a Palaeolithic-type diet. Jönsson *et al.* [38, 39] reported that a Palaeolithic diet is more satiating per calorie than a Mediterranean-like diet. The composition of a diet is likely to be an important factor in satiety and bodyweight management. It has been reported that a high protein content of a diet can increase satiety and weight loss [40, 41]. What aspects of a Palaeolithic-type diet are important in this sense is yet unclear. Spreadbury *et al.* [42] reported that consuming a diet of grain-free whole foods may stimulate gastrointestinal microbiota more consistent with our evolutionary background. All of these factors might explain the apparent favourable effects of the modern “Palaeolithic” diets on satiety and metabolic health.

### Limitations

It can be argued that a two weeks dietary intervention is too short to realize stable effects. Short term positive effects are followed by relapse in many dietary interventions. In our study comparison was made with a single blinded reference diet, rendering our results specific for the Palaeolithic-type diet per se. Importantly, after two weeks 89% of the Palaeolithic group and 64% of the reference group were still motivated to continue their dietary regimes. It will consequently be of utmost importance to study the longer term effects, as Mellberg *et al.* [19] have reported a significant decrease in body weight, fat mass and waist circumference after 6 months, not sustained after 24 months of a Palaeolithic-type diet compared to a reference diet in obese postmenopausal women. Specific aspects of the MetS, as glucose tolerance and probably also other characteristics, might become phenotypically apparent in the longer run, while even greater effects might be seen by allowing ad libitum consumption.

## Conclusions

We conclude that two weeks of a Palaeolithic-type diet, consumed by subjects with characteristics of the MetS, improved several cardiovascular risk factors compared to a healthy reference diet. It proved to be considerably difficult to keep bodyweight stable. Future studies might take full additional advantage of the greater weight loss in the Palaeolithic group by applying an ad libitum study design.

## Abbreviations

BP: Blood pressure
CVD: Cardiovascular disease
DM2: Type 2 diabetes mellitus
DSAT: Differential sugar absorption test
HOMA_IR_: Homeostasis model assessment of insulin resistance
hsCRP: High sensitivity C-reactive protein
MetS: Metabolic syndrome
OGTT: Oral glucose tolerance test.
